# Use of omeprazole, the proton pump inhibitor, as a potential therapy for the capecitabine-induced hand-foot syndrome

**DOI:** 10.1038/s41598-021-88460-9

**Published:** 2021-04-26

**Authors:** Shiori Hiromoto, Takehiro Kawashiri, Natsumi Yamanaka, Daisuke Kobayashi, Keisuke Mine, Mizuki Inoue, Mayako Uchida, Takao Shimazoe

**Affiliations:** 1 Department of Clinical Pharmacy and Pharmaceutical Care, Graduate School of Pharmaceutical Sciences, Kyushu University, Fukuoka, 812-8582 Japan; 2 Education and Research Center for Clinical Pharmacy, Osaka University of Pharmaceutical Sciences, Osaka, 569-1094 Japan

**Keywords:** Cancer, Cancer therapy, Oncology, Cancer therapy, Translational research, Drug safety, Pharmaceutics, Pharmacology, Toxicology

## Abstract

Hand-foot syndrome (HFS), also known as palmar-plantar erythrodysesthesia (PPE), is a major side effect of capecitabine. Although the pathogenesis of HFS remains unknown, some studies suggested a potential involvement of inflammation in its pathogenesis. Proton pump inhibitors (PPIs) have been reported to have anti-inflammatory effects. In this study, we investigated the ameliorative effects of omeprazole, a PPI on capecitabine-related HFS in mice model, and a real-world database. Repeated administration of capecitabine (200 mg/kg, p.o., five times a week for 3 weeks) increased fluid content, redness, and tumor necrosis factor (TNF)-α substance of the mice hind paw. Co-administration of omeprazole (20 mg/kg, p.o., at the same schedule) significantly inhibited these changes induced by capecitabine. Moreover, based on the clinical database analysis of the Food and Drug Administration Adverse Event Reporting System, the group that has used any PPIs had a lower reporting rate of capecitabine-related PPE than the group that has not used any PPIs. (6.25% vs. 8.31%, *p* < 0.0001, reporting odds ratio (ROR) 0.74, 95% confidence interval (CI) 0.65–0.83). Our results suggest that omeprazole may be a potential prophylactic agent for capecitabine-induced HFS.

## Introduction

Hand-foot syndrome (HFS), also known as palmar-plantar erythrodysesthesia (PPE), is a major side effect of antineoplastic drugs such as pyrimidine analogs (e.g., capecitabine), kinase inhibitors, and pegylated liposomal doxorubicin^1–4^. HFS frequently occurs on the palms and soles of the feet and starts with minor skin changes such as erythema and edema, and then, if severe, proceeds to painful edema, blisters, and fissures. Capecitabine is a prodrug of fluorouracil and is used to treat many cancers, including colorectal, gastric, and breast cancers^5–8^. About 50%–68% of patients who take capecitabine develop HFS. Moreover, 11%–24% of these patients have Grade 3 or more severe forms of HFS^9–12^. Besides reduced the quality of life, Grade 2 or more HFS requires discontinuation or reduced dose of capecitabine. Although the pathogenesis of HFS remains unknown, some clinical studies suggested celecoxib a cyclooxygenase-2 (COX-2) inhibitor had a preventive effect for capecitabine-induced HFS^13–15^. Moreover, the inflammatory symptoms, such as edema and reddening of the skin, are reported in capecitabine-induced HFS. The evaluation of the available studies strongly suggests the involvement of inflammation in the development of capecitabine-induced HFS^16^. On the other hand, proton pump inhibitors (PPIs), including omeprazole, are reported to have anti-inflammatory effects in several animal models^17–21^. We hypothesized that omeprazole, with its anti-inflammatory properties, may be effective in preventing or treating HFS. In this study, we investigated the possibility of the proton pump inhibitor omeprazole as prevention for capecitabine-induced HFS in mice model and the clinical database analysis using the Food and Drug Administration Adverse Event Reporting System (FAERS).

## Results

### Effect of omeprazole on HFS induced by capecitabine in mice

**Figure 1:**
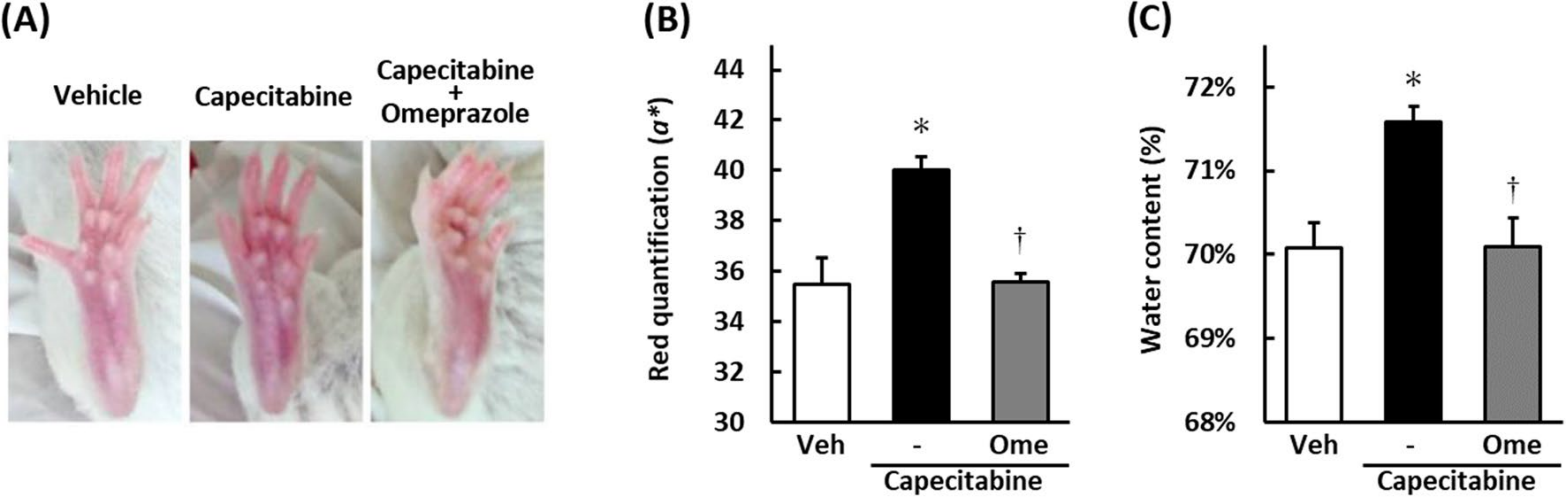
Effects of omeprazole on redness and edema of hind limb plantar induced by capecitabine of the mice. Capecitabine (200 mg/kg) was administered orally five times a week for three weeks. Omeprazole (20 mg/kg) was administered orally in the same way. Photographs of the hind limb plantar of the mice in each drug treatment group on day 19 are shown (**A**). Redness, the intensity of a* in CIE L*a*b* color space, was measured by Image J 1.51 software (**B**). The fluid contents in the plantar skin were measured to assess the edema (**C**). Data are expressed as the mean ± standard error of the mean (n = 5–6).*p < 0.05 compared with vehicle; †*p* < 0.05 compared with capecitabine-alone treatment.

Pictures of the right hind limb plantar of the mice in each drug treatment group on day 19 are shown in Fig. 1A. Redness, a* in CIE Lab (L*a*b* color space), of the plantar, was significantly increased by the repeated administration of capecitabine (200 mg/kg, p.o.) compared with the vehicle treatment on day 19 (*p* < 0.05; Fig. 1B). Co-treatment with omeprazole (20 mg/kg, p.o.) significantly reduced the intensity of the redness induced by capecitabine (*p* < 0.05; Fig. 1B).

The respective administration of capecitabine significantly increases the fluid content of the foot skin compared with the vehicle treatment on the same day (*p* < 0.05; Fig. 1C). Co-treatment with omeprazole significantly inhibited the increase in fluid content induced by capecitabine on day 19 (*p* < 0.05; Fig. 1C).

### Omeprazole reduces inflammatory cytokines in the mouse model of HFS

**Figure 2:**
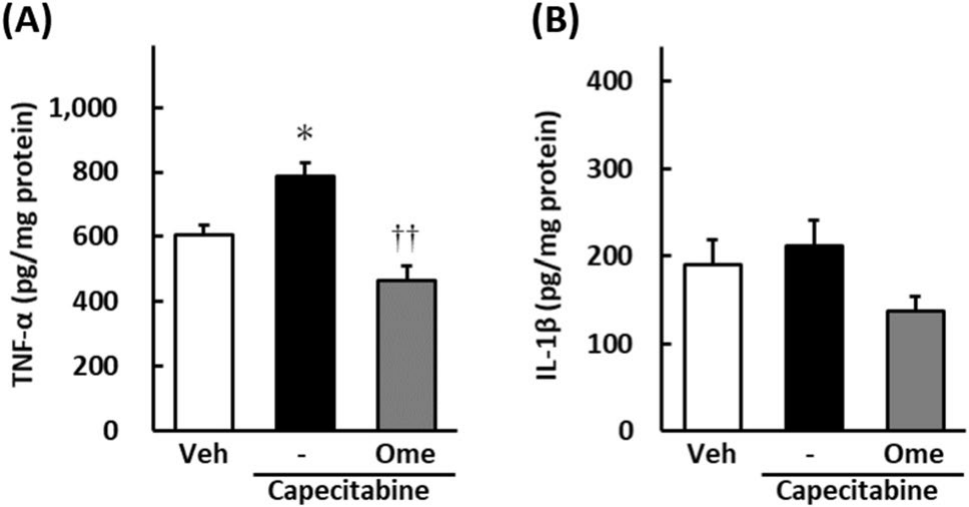
Effects of omeprazole on inflammatory cytokines, TNF-α (**A**) and IL-1β (**B**) in hind limb plantar of the mice. Capecitabine (200 mg/kg) was administered orally five times a week for three weeks. Omeprazole (20 mg/kg) was administered orally in the same way. TNF-α and IL-1β contents were measured by ELISA. Data are expressed as the mean ± standard error of the mean (n = 8–9). ^†^*p* < 0.05 compared with vehicle; **p < 0.01 compared with capecitabine-alone treatment.

Tumor necrosis factor (TNF)-α content of hind limb plantar tissues were significantly increased in the capecitabine-treated group compared to the vehicle group (*p* < 0.05; Fig. 2A). The increase in TNF-α content was significantly inhibited in the omeprazole co-treatment group (*p* < 0.01; Fig. 2A). No significant differences in interleukin (IL)-1β contents were observed between the two groups (Fig. 2B).

### Effects of PPIs on reporting rates of capecitabine-related PPE in FAERS database

**Figure 3:**
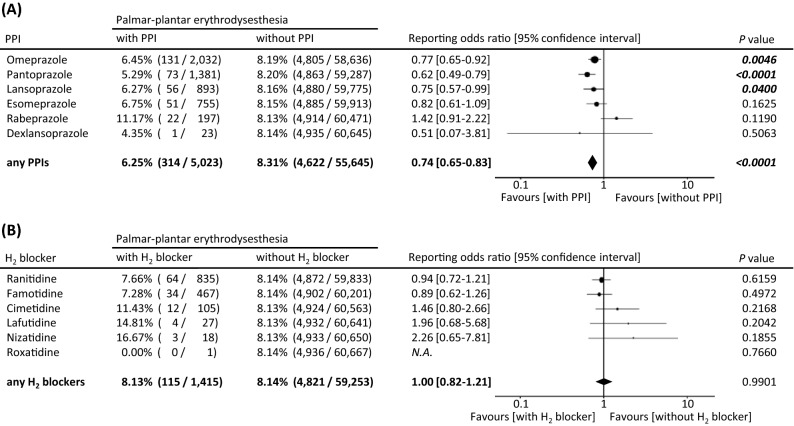
Effects of PPIs (**A**) and H_2_ blockers (**B**) on reporting rates of capecitabine-related PPE in the FAERS database. The report data were extracted using CzeekV Pro (version 5.0.23, INTAGE Healthcare Inc., Tokyo, Japan, accessed August 2020). Of the 12,752,146 adverse event reports from the FAERS between 1997 and the first quarter of 2020, we investigated the reporting rates and the RORs of the palmar-plantar erythrodysesthesia as adverse events after capecitabine use.

Of the 60,668 capecitabine-associated adverse event reports from FAERS database, 4936 reports (8.14%) were PPE. Reporting odds ratios (RORs) of PPE were 0.77 [95% confidence interval (CI) 0.65–0.92], 0.62 [95% CI 0.49–0.79], and 0.75 [95% CI 0.57–0.99] in patients with omeprazole, pantoprazole, and lansoprazole, each respectively (Fig. 3A). The patients using any PPIs had a significantly lower reporting rate of PPE compared to the patients without any PPIs (6.25% vs. 8.31%, *p* < 0.0001, ROR: 0.74 [0.65–0.83]; Fig. 3A). In contrast, no significant difference in the reporting rate of PPE was observed between groups with and without any H_2_ blockers (8.13% vs. 8.14%, *p* = 0.9901, ROR = 1.00 [0.82–1.21]; Fig. 3B).

## Discussion

The pathogenesis of capecitabine-induced HFS is still unknown. However, some studies indicate the involvement of inflammation in its pathogenesis^13–16^. In the present study, we evaluated the effect of omeprazole, a drug reported to have anti-inflammatory effects in the mouse model of capecitabine-induced HFS. Edema and erythema are the main symptoms of HFS. Therefore, we analyzed fluid content as an indicator of edema and redness as an indicator of erythema in a mouse model. Omeprazole treatment reduced edema and redness in the feet, which suggests omeprazole may exert a skin protective effect against capecitabine.

The same model was used to investigate the mechanism of action of how capecitabine exerts the skin protective effect by measuring the inflammatory cytokines productions from the skin of the feet. Omeprazole inhibited capecitabine-induced increases of the cytokine productions (IL-1β and TNF-**α**) from the skin. This observation suggests that anti-inflammatory effects may be involved in the protective effect of omeprazole on the skin. Omeprazole and esomeprazole, the S-isomer of omeprazole, have been reported to have anti-inflammatory effects. Moreover, some studies have reported esomeprazole to inhibit TNF-α and IL-1β and other inflammatory molecules such as IL-6, vascular cell adhesion molecule-1, and inducible nitric oxide synthase, which may contribute to the skin protective effect of omeprazole^17,20,22,23^.

Additionally, we investigated the effect of omeprazole on capecitabine-induced HFS in the clinical setting using the real-world database, FAERS. Besides omeprazole, other PPIs, including pantoprazole and lansoprazole, significantly suppressed the reporting rate of PPE. Moreover, the reporting rate of PPE in the group with any PPIs was also lower than it without any PPIs (6.25% vs. 8.31%, *P* < 0.0001, ROR = 0.74, 95% confidence interval (CI) 0.65–0.83). In sum, the anti-HFS action may be a class effect common to all PPIs. The effects of PPIs other than omeprazole were not tested in our animal model. Lugini and colleagues have reported that the potency of the action of each PPI depends on the hydrophilicity or lipophilicity of the PPI structures^24^. Thus, the difference in the potency of the effects on HFS needs to be examined in the future. Meanwhile, despite H2 blockers to have an inhibitory effect on gastric acid secretion like PPIs, the use of any H_2_ blockers did not have any effects on the reporting rate of PPE. Thus, effects other than the gastric acid suppression may be involved in the ameliorative effect of PPI on capecitabine-related HFS.

The effect of omeprazole on the antitumor effects of capecitabine was not examined in this study. Some reports suggest that omeprazole induces multidrug resistance-associated protein (MRP3), or known as ATP binding cassette subfamily C member (ABCC3) in the liver^25^. Pantoprazole, a drug in a class PPIs, has reported to suppress the MRP1 (ABCC1) signaling pathway and reduce cell survival in drug-resistant gastric adenocarcinoma cells^26^. Further studies should be required to determine the effect of omeprazole on the antitumor effects of capecitabine.

There are a series of clinical studies on the use of PPIs in the treatment of cancer patients^27–32^. Some of them are retrospective studies showing that PPIs may increase the efficacy of chemotherapy^30^ or prevent cancer^31,32^. Moreover, PPIs have also been reported to enhance the antitumor activity of carbonic anhydrase IX inhibitors and reverse transcriptase inhibitors in basic studies^33,34^. Furthermore, there is a general agreement on the possibility to repurpose PPIs in cancer treatments^35–37^. On the other hand, several clinical studies have recentry reported that gastric acid secretion inhibitors, including PPIs and H2 blockers, attenuate the effect of capecitabine^38–41^. Further information on the efficacy and safety of the combination of capecitabine and PPIs in cancer patients needs to be established. Nevertheless, the present study suggests that co-administration of omeprazole with capecitabine may prevent capecitabine-induced HFS in both animal models and clinical database analysis at least.

## Methods

### Animals

We used ICR mice (six-week-old, 30–35 g, Japan SLC, Inc., Shizuoka, Japan) for the experiments. Mice were bred in groups of 4–5 per cage, with a 12:12-h light–dark cycle. Animals were fed water and food ad libitum. All animal experiments were approved by the Experimental Animal Care and Use Committee of Kyushu University and conducted according to the guidelines of the National Institutes of Health. And all experiments were carried out in compliance with the ARRIVE guidelines.

### Drugs

In the capecitabine-induced HFS model, capecitabine (200 mg/kg, Fluorochem, Ltd., Derbyshire, UK) or a vehicle (5% methylcellulose; MP Biomedicals, CA, USA) was administered orally (p.o.), five times a week for three weeks (days 1–5, 8–12, and 15–19). Omeprazole (20 mg/kg, FUJIFILM Wako Pure Chemical Corporation, Osaka, Japan) was administered orally (p.o.) at the same schedule. Each drug was administered at a volume of 10 mL/kg. The doses of these drugs were determined based on previous reports^42–44^.

### Assessment of HFS in mice

The soles of the mouse's feet were photographed to assess the redness of the foot skin. The a*-intensity of the CIE L*a*b* color space of the captured images was measured by Image J 1.51 software (Wayne Rasband, National Institutes of Health, MD, USA). The skin of the hind limb plantar was collected to assess the edema. The fluid contents were calculated by Eq. (1).

1 $$  {\text{Fluid}}\;{\text{content}}(\% ) = \left( {1 - \frac{{{\text{weight}}\;{\text{dry}}}}{{{\text{weight}}\;{\text{wet}}}}} \right) \times 100 $$

In this formula, weight _wet_ refers to sample weight immediately after the collection; weight _dry_ refers to sample weight after drying at 65 °C for 48 h. Each test was conducted on the last day of the experiment (day 19).

### Quantification of IL-1β and TNF-α in mice hind limb plantar

On day 19, the hind limb plantar tissue of the mice was collected and homogenized in buffer containing 1 mM phenylmethylsulfonyl fluoride (FUJIFILM Wako Pure Chemical Corporation,), and the content of IL-1β and TNF-α were analyzed using a commercial ELISA kit (Proteintech Group, Inc., IL, USA). The procedure followed the instructions of the kit.

### Analysis of FAERS data

To investigate the efficacy of PPIs, including omeprazole, in reducing the incidence of HFS in a clinical setting, we analyzed the incidence of HFS in patients taking capecitabine using the FAERS database. The report data were extracted using CzeekV Pro (version 5.0.23, INTAGE Healthcare Inc., Tokyo, Japan, accessed August 2020). Of the 12,752,146 adverse event reports from the FAERS between 1997 and the first quarter of 2020, we investigated the reporting rates and the RORs of PPE as adverse events after capecitabine use. The RORs and 95% CIs were calculated by the Eqs. (2) and (3), respectively^45^.

2 $$   {\text{ROR}} = \frac{{{\text{n}}11{\text{ / n}}21}}{{{\text{n}}12{\text{ / n}}22}} $$

3 $$  95\% {\text{CI}} = {\text{exp}}\left[ {\log \left( {{\text{ROR}}} \right) \pm 1.96\sqrt {\frac{1}{{{\text{n}}11}} + \frac{1}{{{\text{n}}12}} + \frac{1}{{{\text{n}}21}} + \frac{1}{{{\text{n}}22}}} } \right]  $$

In these formulas, n11 refers to patients who used PPIs or H_2_ blockers as a reference and reported the PPE; n12 refers to patients who used PPIs or H_2_ blockers but did not report the PPE; n21 refers to patients who did not use these drugs but reported the PPE, and n22 refers to patients who did not use these drugs and did not report the PPE.

### Statistical analysis

The results are expressed as the mean ± standard error of the mean. Statistical analyzes were performed using a one-way analysis of variance followed by the Tukey–Kramer test and a chi-square test for the animal experiments and the FAERS analysis, respectively (Statview; Abacus Concepts, Berkeley, CA, USA). A probability level of *p* < 0.05 was accepted as statistically significant.

## Data Availability

The data that support the findings of this study are available from the corresponding author upon reasonable request.
